# The evolution of cerebellum structure correlates with nest complexity

**DOI:** 10.1098/rsbl.2013.0687

**Published:** 2013-12-23

**Authors:** Zachary J. Hall, Sally E. Street, Susan D. Healy

**Affiliations:** School of Biology, University of St Andrews, Harold Mitchell Building, St Andrews KY16 9TH, UK

**Keywords:** nest construction, cerebellar foliation, avian cerebellum

## Abstract

Across the brains of different bird species, the cerebellum varies greatly in the amount of surface folding (foliation). The degree of cerebellar foliation is thought to correlate positively with the processing capacity of the cerebellum, supporting complex motor abilities, particularly manipulative skills. Here, we tested this hypothesis by investigating the relationship between cerebellar foliation and species-typical nest structure in birds. Increasing complexity of nest structure is a measure of a bird's ability to manipulate nesting material into the required shape. Consistent with our hypothesis, avian cerebellar foliation increases as the complexity of the nest built increases, setting the scene for the exploration of nest building at the neural level.

## 1. Introduction

All vertebrates have a brain structure called the cerebellum. Historically, the cerebellum was considered to play a major role in motor control [1] but is now known also to be involved in a range of cognitive processes, including learning, memory and language in humans [2]. Two striking morphological features of the cerebellum are the variation in both its volume and foliation (amount of surface folding) across species: amphibians and reptiles have unfolded cerebella, whereas birds and mammals have variably convoluted cerebella [3,4]. It has been suggested that, in birds at least, increased cerebellar foliation increases the density of cerebellar neural circuitry, allowing increased processing capacity and enhanced motor abilities, specifically manipulative skills [5,6]. Some support for this suggestion is provided by the positive correlation between cerebellar foliation and tool use in birds [6] and between cerebellum volume and extractive foraging in primates [7] and neural activation (as seen by functional imaging) in the cerebellum during tool use in monkeys [8].

Nest building in birds also requires some manipulative skills, which vary depending on the complexity of the nest built. Here, we examined whether variation in cerebellar foliation index (CFI; [4]) in birds is explained by the variation in the complexity of their species-typical nest structure. We predicted that species that build more structurally complex nests would have higher CFIs than would species that build simpler nests.

## 2. Material and methods

### (a) Cerebellar foliation and nest structure

We collected data on CFI, measured as the degree of cerebellar cortex folding compared with a hypothetical unfolded cortex for the same cerebellum size, cerebellum volume, whole brain volume and body mass from Iwaniuk *et al*. [4] for 87 bird species.

We then gathered descriptions of the species-typical nest structure from published studies and texts (see electronic supplementary material, table S1). Based on these descriptions, we categorized nest structures as No nest, Platform, Cup, Domed and Excavation nests. Birds that do not excavate or construct a nest but lay eggs directly on a bare substrate or in a nest built by another species were categorized as building no nest. Platform nests are unshaped piles of collected nesting material, including material used to line ground scrapes and depressions. Cup nests have nest walls created during construction by the bird and not by depression of the nest's centre by the weight of the bird and eggs’ during incubation. Domed nests have both nest walls and a roof. Finally, excavation nests are tunnels or chambers dug using the beak or feet into a substrate. Unlike Hansell [9], we did not differentiate between platform nests built in the tree and those on the ground (referred to as ‘plate’ and ‘bed’ nests, respectively, in [9]) but we did differentiate between species that excavate nests and those that nest in natural cavities or cavities excavated by other species (both referred to as ‘cavity nests’ in [9]). These differences in nest categorization reflected our focus on the motor processes involved in constructing the nest structure, regardless of nest location or materials used.

We focused on comparing no nest, platform and cup nest structures because these three nest structures differ in the degree to which material is collected and manipulated during construction: birds building no nest do not collect or manipulate nest material, platform nests require the collection but little manipulation of material while cup nests require collection and manipulation of nest material to produce walls in the cup structure. Because excavation behaviour is difficult to compare with the collection and manipulation of nest material, we excluded species that built excavation nests from further analysis. Furthermore, because only two species (*Acanthiza pusilla* and *Menura novaehollandiae*) in our sample constructed domed nests, we also excluded these species from analysis as well as those species without a nest description. After these exclusions, 64 species remained in our analysis (see electronic supplementary material, S1). Keywords used to categorize species-typical nest structure are summarized in table 1.

**Table 1.** Terminology in published nest descriptions used to classify species-typical nest structure. In our nest structure classification, we focused on the behaviours involved in collecting and manipulating nest material as well as manipulating nesting substrates, irrespective of nest location or the materials used.

nest classification	terminology in literature
no nest	no evidence of construction/excavation
	cavity excavated by other species
	nestbox
	tree hollow/hole
	unlined scrape
	nest on bare ground
	no nest/no nesting material
	old stick nest of other species
	shallow knot-hole
platform	platform
	lined scrape/depression
	saucer-shaped
	bed of material
	pile of material
	mud nest
cup	bowl
	cup
	cup-shaped
	half cup

### (b) Statistical methods and analyses

To account for the statistical non-independence of datasets including multiple species, we analysed data using the phylogenetic generalized least squares (PGLS) approach, which incorporates phylogenetic relatedness of species into the error term of a regression model [10]. Regression analysis included nest structure as an independent variable on three levels (no nest, platform and cup) and CFI as a continuous, dependent variable. To account for allometric scaling effects on CFI, we included cerebellum volume as a covariate. Cerebellum volume was log-transformed to achieve normality (Shapiro–Wilkes test, *p* > 0.05). Although previous CFI analyses included other allometric variables (body size, whole brain volume and whole brain–cerebellum volume [4]), we found that cerebellum volume predicted CFI better than the other allometric measures and after including cerebellum volume as a covariate no other allometric variable explained significant variation in CFI. In addition to testing the main effect of nest structure, we also made three planned contrasts (no nest versus platform, no nest versus cup, and platform versus cup) by changing which factor level was the reference level in the model. We ran analyses in R ([11]) using the packages ape [12] and caper [13] and viewed trees in FigTree [14] and DensiTree [15].

**Figure 1.**
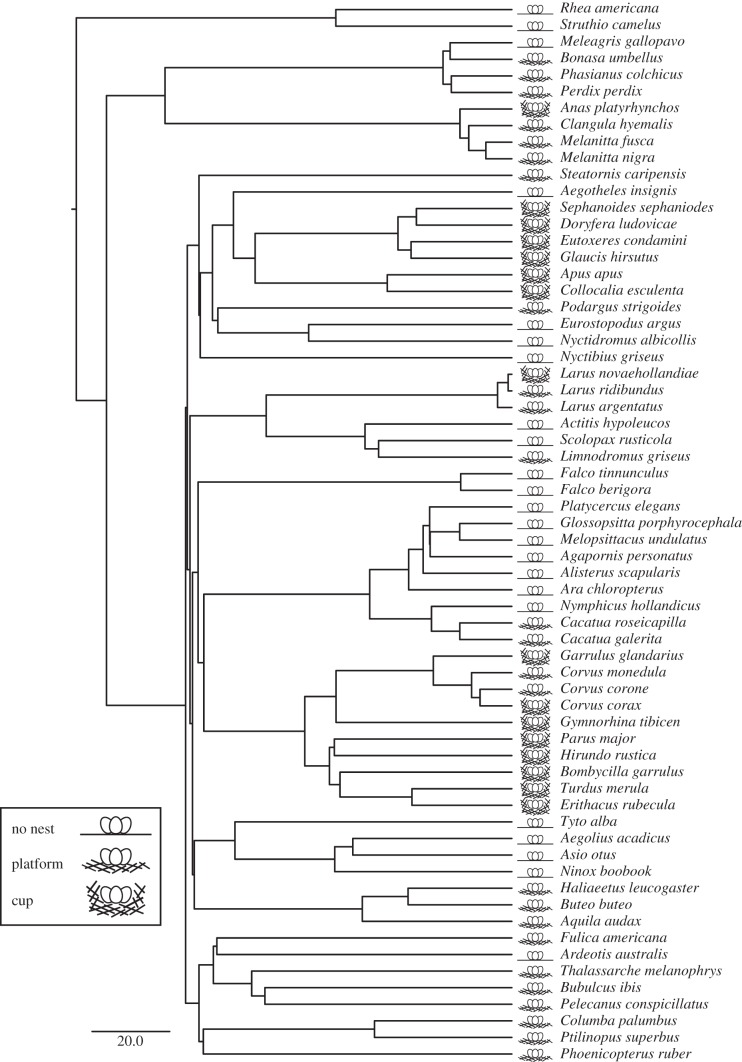
Sample phylogeny of bird species included in regression analysis and species-typical nest structure classification. We included species from [6] that had a description of the typical nest structure we could classify as no nest, a platform or a cup. Branch lengths represent time. Scale bar represents 20 Myr [17]. Species names taken from [17].

To account for phylogenetic uncertainty, we ran our PGLS models across a sample of 3000 phylogenies built using a family backbone by Hackett and co-workers [16,17] with restricted phylogenetic signal estimation (*λ* = lower: 0.01–0.1, upper: 0.95–0.99). We used model averaging (following [18]) to estimate average parameters from PGLS regressions across the tree-block, weighted by the probability of the model given each tree. Main effects could not be model-averaged across the tree-block because they are calculated from comparison of models with and without nest structure using ANOVA. Instead, we present the minimum *F* and maximum *p* values reported across the tree-block as a conservative means of testing for the main effect across varying phylogenies. *λ* was fixed at either 0.85 or 0.95 when testing for main effects. In order to ensure that our results were not affected by model uncertainty in addition to phylogenetic uncertainty, we re-ran our main PGLS analyses using Bayesian Markov chain Monte Carlo methods, which account for both model and phylogenetic uncertainty (see electronic supplementary material, S2). All bird phylogenies were acquired from www.birdtree.org [17]. An example phylogeny is presented in figure 1.

## 3. Results

**Figure 2.**
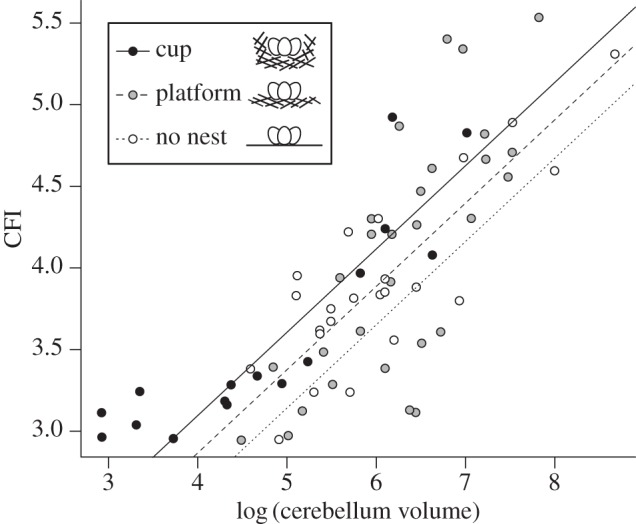
Regression lines between log-transformed cerebellum volume and CFI of bird species that build cup, platform and no nest. Dots represent log-transformed cerebellum volume and CFI for bird species that build cup (black), platform (grey) and no nest (white). Slopes and intercepts for all three groups were estimated from PGLS regression models. For a given cerebellum volume, species that build cup nests have significantly more foliated cerebella than do species that build platform nests and no nest (both *p* < 0.05) and species that build platform nests have significantly more foliated cerebella than species that build no nest (*p* < 0.05).

Across 64 species of bird, nest structure is significantly associated with cerebellar foliation (*F*_1,60_ > 3.875, *p* < 0.026, *R*^2^ = 0.615; using *λ* = 0.85 = model-averaged estimate from main regression model). Specific contrasts confirm our predictions: species that build a platform nest have higher CFIs than do species that do not build nests (figure 2; *t* = 2.047, *p* = 0.047), species that build a cup nest have higher CFIs than species that do not build nests (figure 2; *t* = 3.165, *p* = 0.003) and species that build a cup nest have higher CFIs than species that build a platform nest (figure 2; *t* = 2.020, *p* = 0.049). Altogether, as nests increase in structural complexity (no nest → platform → cup), CFI also increases. Furthermore, nest structure specifically explained variation in CFI when we used log-transformed cerebellum volume as a covariate and not other allometric variables (see electronic supplementary material, S2).

## 4. Discussion

Bird species that build more structurally complex nests have greater cerebellar foliation than do species that build simpler nests, which supports the hypothesis that increased cerebellar foliation enables enhanced manipulative motor skills [5]. Similarly, Yopak *et al*. [19] suggest that CFI might correlate with increasingly sophisticated behaviours in chondrichthyes, for example agile capture of cephalopod prey in the tawny nurse shark (*Nebrius ferrugineus*). In conjunction with these data on chondrichthyes, our findings suggest that increasing cerebellar foliation may be a mechanism that is conserved across vertebrates allowing improved motor control and increasingly complex behaviours. Such an increase in foliation may underpin the positive correlation between cerebellum volume and extractive foraging in primates [7]. In birds at least, increased cerebellar foliation is hypothesized to increase the density of Purkinje cells, the predominant neuron in the cerebellar cortex and only source of cerebellar output, which is thereby thought to increase the processing capacity of the cerebellum [6]. Although here we found that cerebellar foliation is associated with manipulative skill, other processes involved in nest construction behaviour that are also supported by the cerebellum, such as motor sequencing and learning, may also explain the correlation between nest complexity and cerebellar foliation. Functional studies correlating neural activity with nest construction behaviour may help to identify which of the processes associated with nest construction involve the cerebellum.

In our analyses, we used a much simpler nest classification system relative to those used previously [9] to examine causes of variation in nest building. For example, we excluded nesting materials, nest attachment to substrates and nest location. By doing so, however, we had a dataset that was amenable to current comparative statistical analytical techniques. The association between variation in CFI and in nest structural complexity that we show here would suggest that this simple classification system may be useful for further investigation of the evolution of nest design.

Here, we found that cerebellar foliation is associated with differences in nest construction behaviour in birds. Across all bird species, nest construction behaviour varies tremendously, beyond the three nest classifications we tested here [9]. By continuing to identify the neural underpinnings of nest construction, we can take advantage of this variation in species-specific behaviour to understand how evolution has shaped the brain to produce unique behaviours.

## Data accessibility

Nest classification, nest structure description sources and neuroanatomical measures are all included in the electronic supplementary material, S1.
